# Valorisation of “La Palma” Volcanic Ash for Making Portland-Blended, Alkaline and Hybrid Portland–Alkaline Cements

**DOI:** 10.3390/ma17010242

**Published:** 2024-01-02

**Authors:** Pablo Martín-Rodríguez, Ana Fernández-Jiménez, María del Mar Alonso, Angel Palomo, Inés García-Lodeiro

**Affiliations:** 1 Instituto Eduardo Torroja (IEtcc), CSIC, C/Serrano Galvache 4, 28033 Madrid, Spain; pablo.martin@ietcc.csic.es (P.M.-R.); mmalonso@ietcc.csic.es (M.d.M.A.); palomo@ietcc.csic.es (A.P.); iglodeiro@ietcc.csic.es (I.G.-L.); 2 Campus de Leganés, Universidad Carlos III de Madrid, Avda. Universidad, 30, 28911 Madrid, Spain

**Keywords:** volcanic fly ash, blended cement, hybrid alkaline cement, alkaline cement

## Abstract

The present work evaluates the feasibility of using volcanic fly ash (VFA) generated by the eruption of the Tajogaite volcano on the island of La Palma (Spain) in 2021, as a precursor in the preparation of cementitious materials with different Portland cement (PC) replacement levels (0%, 30%, 70% and 100%), in the absence (Blended Cement, BC) and presence of an alkaline activator (Hybrid Alkaline Cement, HAC, and Alkaline Cements, AC). Hydration kinetics (isothermal conduction calorimetry), paste mechanical strengths and reaction products were characterised by XRD, FTIR, TG/DTG and BSEM/EDX. The results obtained indicate that the strengths developed by the hybrid alkaline cements (HAC) are higher than those of the blended cements (BC), especially at the age of 2 days, where 25 MPa were obtained with the replacement of 70% PC by VFA. Alkaline cements (AC, 100% VFA) that were prepared with 8 M NaOH solution as the activator reached 40 MPa after 2 days. It was observed that in all the binders, depending on the initial composition of the binder mixture and the percentage of replacement and/or activator, VFA reacts to form cementitious gels, C-A-S-H and N-A-S-H type, which supports its use as a mineral addition to blended cement or as a precursor in the preparation of alkaline and hybrid alkaline cements.

## 1. Introduction

**Figure 1:**
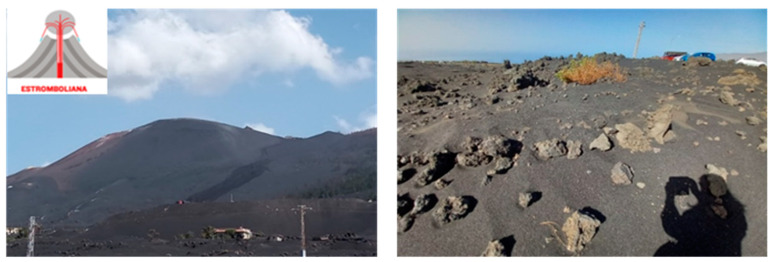
Landscape generated after the eruption of the Tajogaite volcano (La Palma, Spain).

From September to December 2021 (a period of 85 days), the Tajogaite volcano (Montaña Rajada) erupted in the Cumbre Vieja area of the island of La Palma, Spain [1,2]. The volcano, which was of a strombolian type, had several craters and ejected a large amount of pyroclastic material (hot mixture of gases, ash and rock fragments). Among these materials, volcanic fly ash (VFA) is formed by the rapid cooling of small particles of molten material inside the volcano in contact with the air. These VFA particles have been transported by volcanic gases and are deposited as a powdery layer on the ground (see Figure 1). The thickness of the ash layer and the size of the ash particles falling to the ground depend on the distance from the volcano, with the smaller particles travelling greater distances, so the size distribution of the ash particles can vary greatly in different affected areas of the island.

Volcanic fly ashes (VFAs) generally have a high vitreous phase content due to their rapid cooling and are mainly composed of silica and alumina (≥70% by weight) [3], which gives them a pozzolanic behaviour, although they also have relevant contents of Fe_2_O_3_ and MgO [4]. Recently, several reviews have investigated the chemical and mineralogical characteristics of different VFAs and their reactivity as a function of their origin (geographical location and volcanic type) [3,5,6].

VFAs have been used in the development of binders since ancient times [7,8] because, as mentioned above, they are considered to be natural pozzolans [9]. Pozzolans are materials that can combine with lime at room temperature in the presence of water to form hydration products with binding properties [10]. There are many studies in the literature [5,11,12,13,14,15,16] analysing the use of VFAs as supplementary cementitious materials (SCMs) in cements and concretes. These studies suggest a level of Portland cement replacement in mortars and concretes between 20 and 30%. Higher replacements result in a significant reduction in mechanical properties at early ages compared to the reference cement. The reason for this is that the pozzolanic reactions are slower than those corresponding to the hydration of Portland cement. Some of the main advantages of using VFA as a replacement for cement (blended cement, BC) are the use of lower clinker contents, which leads to a reduction in production costs and a reduction in CO_2_ emissions. Some technical advantages are also achieved, such as increased resistance to sulphate and chloride attacks due to refinement of the microstructure [11]. In the pozzolanic reaction, the VFAs react with Ca(OH)_2_, a secondary phase of cement hydration, to form mainly C-S-H or C-(A)-S-H gels, which causes a densification of the porous structure of the pastes [17,18]. With regard to the use of VFA from the La Palma volcano, Rosales et al. [19] demonstrated that these ashes could be used as SCMs with substitution levels of 25%, concluding that it is necessary to carry out a grinding process to reduce the particle size of the VFA, in order to improve its pozzolanic behaviour. The particle size of the VFA can be modified by sieving to remove the sandy fraction or by grinding to a finer particle size [4].

Several studies [3,20,21,22,23,24] have shown that VFAs can also potentially be used as a precursor in the production of alkali-activated cements (AC). It is considered that VFAs are suitable for use as a precursor when they have a composition of SiO_2_ + Al_2_O_3_ ≥ 70%, a SiO_2_/Al_2_O_3_ molar ratio between 3.3 and 4.5 and an amorphous phase content >36%. These papers also indicate several options for increasing the reactivity of VFA in ACs. Some of these recommendations are (i) mechanical activation by grinding; (ii) thermal activation by calcination; (iii) chemical activation with high alkaline activators; (iv) thermal curing of pastes, mortars or concretes (curing temperature between 40 and 90 °C); and (v) preparation of mixed alkaline cements by adding other reactive materials rich in SiO_2_, Al_2_O_3_ and CaO (e.g., metakaolin or other calcined clays, blast furnace slag, fly ash from coal combustion, etc.), which significantly improves the properties of the final binder.

In this field of research, it is worth mentioning the recent study published by Tashima et al. in 2023 [2], where VFAs from the “Cumbre Vieja” volcano (Island of La Palma-Spain) were used in the production of alkaline cements, using a sodium silicate solution and curing temperatures between 65 and 85 °C. In this work, compressive strength values of up to 80 MPa were obtained in pastes, with the main reaction product being an N-A-S-H type gel [25,26,27,28].

The aforementioned studies show that VFAs can be used in the production of both blended cements and alkaline cements, although there are still challenges and unresolved issues to be addressed. In blended cements, the level of replacement of VFAs by cement generally does not exceed 20–30%. In alkaline cements, 100% replacement can be achieved, but this requires the use of alkaline activators with relatively high alkalinity and high energy and environmental costs (NaOH/KOH/Na_2_SiO_3_…). A more sustainable and economical option would therefore be the production of hybrid alkaline cements. On the one hand, the degree of Portland cement substitution could be increased up to 70%, thus improving the degree of PC substitution in blended cements, and alternative alkaline activators (green activators [29,30]) could be used with less environmental and economic impact.

In this line, this article evaluates the potential of using the volcanic fly ash (VFA) generated by the eruption of the volcano of the island of La Palma in 2021 as a precursor in the elaboration of binders with different Portland cement replacement levels of 0%, 30%, 70% and 100%, in the absence and presence of an alkaline activator, which increases the reactivity of the VFA.

## 2. Material Characterisation and Methodologies

### 2.1. Chemical, Mineralogical and Physical Characterisation of the Raw Materials

The materials used in this investigation were (i) the aforementioned volcanic fly ash (VFA) and (ii) a commercial cement CEM I 52.5R (PC). The chemical analysis of the starting materials (see Table 1) was carried out by X-ray fluorescence (XRF) (S8 Tiger Bruker, from IETcc, Madrid, Spain). The volcanic ash has SiO_2_ as the main oxide, followed by Fe_2_O_3_, Al_2_O_3_, CaO and MgO, with minor contents of alkaline oxides. These values are similar to those obtained by other authors [2,19]. In the case of PC, as expected, the main oxides were CaO, SiO_2_ and Al_2_O_3_.

**Figure 2:**
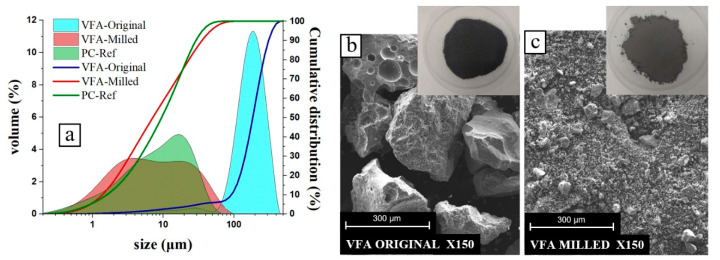
(**a**) Particle size distribution of both PC, original VFA and VFA after milling; (**b**) SEM photograph of original VFA; and (**c**) SEM photograph of milled VFA.

Raw volcanic ash is characterised by a metallic black colour (see Figure 2) and a particle size similar to fine sand, with more than 90% of the particles larger than 200 microns. To increase its reactivity, the volcanic ash was dried at 100 °C and then ground in a ball mill (500 g batch/batch, 5 h) until 96% of the particles passed the 45-micron sieve, and 91% of the particles were below 32 microns. Figure 2a shows the particle size distribution (determined by laser diffraction using the Mastersizer S. Malvern, from IETcc, Madrid, Spain) of the VFA before and after grinding, as well as that of the reference PC.

Figure 2b,c show SEM micrographs of both raw and milled VFA. The volcanic ashes show a compact angular particle morphology. This morphology is clearly different from that of fly ash from coal-fired power plants (spherical particles [31]). Their morphology is similar to that of blast furnace slag particles [32]).

Milled VFA was used in this work. The mineralogical analysis of VFA and PC was carried out by XRD in a BRUKER-AXS D8 ADVANCE diffractometer (from IETcc, Madrid, Spain). The recording was carried out using Cu-Kα1 radiation, in the range between 2θ values of 5–60° with a step/size equal to 0.019736° and a time/step of 0.5 s. The diffractograms were analysed using the Difrac. Plus, EVA V4.2 software.

**Figure 3:**
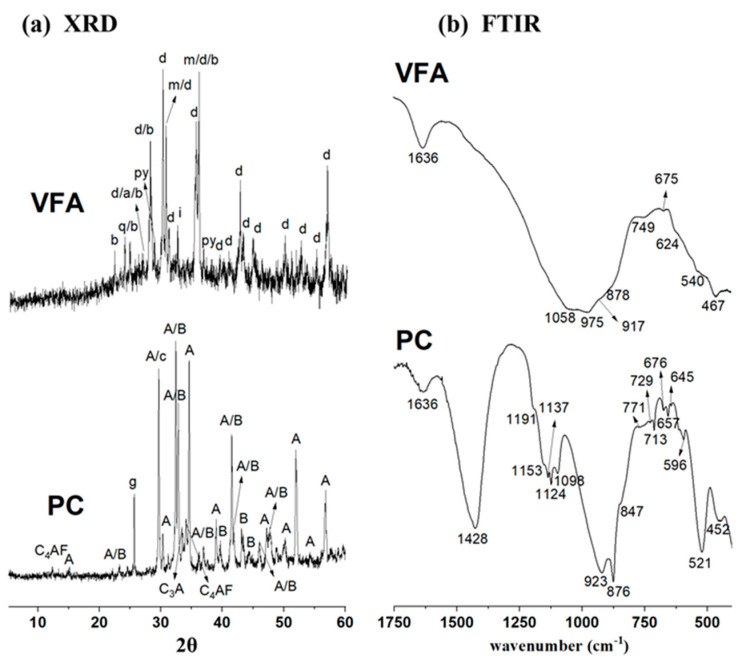
(**a**) XRD patterns and (**b**) FTIR spectra of Portland cement (PC) and the volcanic fly ash (VFA). Legend: d: diopside (MgCaSi_2_O_6_) (COD 9004319); a: augite (Ca,Mg,Fe)_2_(Si,Al)_2_O_6_ (COD 9006247); q: cristobalita (SiO_2_) (COD 9001578); m: magnetite (Fe_2_O_3_) (COD 9006247); b: bytownite (Ca,Na)(Si,Al)_4_O_8_) (COD 9011200); i: ilmenite (FeTiO_3_) (COD 9000910); py: pyroxene (Fe_0.44_Mg_0.56_SiO_3_) (COD 9001577); A: alite (3CaO·SiO_2_) (COD 1540705)); B: belite (2CaO·SiO_2_) (COD 9012793)); C_3_A: tricalcium aluminate (3CaO·Al_2_O_3_) (COD 9014359)); C_4_AF: ferritic phase (4CaO·Al_2_O_3_·Fe_2_O_3_) (COD 9015955); g: anhydrite (CaSO_4_) (COD 5000040); c: calcite (CaCO_3_) (COD 9016022).

Figure 3a shows the XRD pattern corresponding to the VFA sample, where the presence of a localised halo at 2θ values between 20 and 50, associated with the amorphous component of the ash, is observed. Several peaks associated with different crystalline phases, mainly cristobalite (SiO_2_), diopside (MgCaSi_2_O_6_), augite (Ca,Mg,Fe)_2_(Si,Al)_2_O_6_, magnetite (Fe_2_O_3_), bytownite (Ca,Na)(Si,Al)4O_8_ and ilmenite (FeTiO_3_) were also identified. Diopside and augite are Mg and Al silicates that can form solid, dark green or black solutions. Bytownite, on the other hand, is a calcium-rich mineral of the plagioclase solid solution series. Ilmenite, which is mainly composed of Fe and Ti, is an iron black with a metallic sheen, a product of magmatic segregation, and is associated with crystals such as magnetite or rutile. The presence of these minerals is responsible for the black colour of the volcanic ash used. The mineralogical analysis of the PC (see Figure 3a shows the phases corresponding to clinker: alite (3CaO-SiO_2_) and belite (2CaO-SiO_2_), tricalcium aluminate (3CaO-Al_2_O_3_) and ferrites (4CaO-Al_2_O_3_-Fe_2_O_3_). Peaks corresponding to anhidrite (CaSO_4_) and traces of calcite (CaCO_3_) are also detected.

A Thermo Scientific NICOLET 600 FT-IR spectrophotometer (from IETcc, Madrid, Spain) was also used to characterise the starting materials. Solid samples were prepared by mixing 0.01 g of material with 0.2 g of KBr, grinding and homogenising the sample and compacting the powder into pellets by uniaxial pressure. Spectra were recorded between 4000 and 400 cm^−1^ (“mid-IR”).

Figure 3b show the FTIR spectra of VFA and PC. The VFA shows a very broad and asymmetric main band located around 1000 cm^−1^, where different shoulders (1058 cm^−1^ and 975 cm^−1^) can be distinguished. This band, usually attributed to the asymmetric stretching vibrations of the T-O bonds (where T is Si or Al) [33,34], is the result of the superposition of both the amorphous phase of the ash containing this type of bond and the crystalline phases, diopside, augite and bytownite, identified by XRD. In the PC spectrum, the bands associated with calcium silicates (923, 521 and 452 cm^−1^), those associated with sulphates (1153, 1118, 1098, 664 and 596 cm^−1^) and the bands associated with carbonates, previously identified by XRD (1428, 876 and 713 cm^−1^) are clearly identified.

In order to assess the possible pozzolanic behaviour of the VFA and its potential use as an addition in cements, the saturated lime dissolution test was carried out [35,36]. The results obtained after 2 and 28 days indicate that the VFA fixes CaO and therefore shows pozzolanic activity (Table 2). The potentially reactive SiO_2_ and Al_2_O_3_ content of the VFA was evaluated by a chemical attack with 1% HF [37,38]. The results are also shown in Table 2.

### 2.2. Preparation of the Pastes

Cement (PC) and volcanic fly ash (VFA) were used to produce different types of binders: the nomenclature and dosification used in the different cements, the percentages of ash and PC and the liquid used for hydration (8 M NaOH solution for 100% alkaline cements (without PC) and water for the rest of the binders) and the presence of the alkaline activator of moderate alkalinity are shown in Table 3.

CEM, BC and HAC pastes were prepared by simply adding water to the solid mixture and curing for 20 h at 25 °C and 99% RH. A mixture of 3% Na_2_SO_4_ + 2% CaSO_4_ was used as a moderately alkaline solid activator for the HAC pastes. To prepare the pastes of the 100% alkaline cements (AC), the VFA was mixed with an 8 M NaOH solution, and the resulting pastes were cured for 20 h at 85 °C and 99% RH and then stored in the climatic chamber (21 °C, 99% RH) until the test age.

Prismatic specimens of 1 × 1 × 6 cm^3^ were prepared from all the pastes and characterised at different ages from a mechanical, mineralogical and microstructural point of view. At the testing ages, the samples were ground, and the hydration reactions were stopped with isopropanol for 24 h and then stored in a desiccator for 7 days.

### 2.3. Mechanical, Mineralogical and Microstructural Characterisation of Pastes

The mechanical compression characterisation was carried out at 2 and 28 days, using an Ibertest Autotest 200/-10-SW press (from IETcc, Madrid, Spain), obtaining 6 replicas per series of samples. The mineralogical and microstructural characterisation was carried out on the replicas, except for the scanning electron microscopy study, for which an untested specimen was used.

Mineralogical characterisation of the reaction products was carried out by X-ray diffraction (XRD, from IETcc, Madrid, Spain) using the diffractometer mentioned in Section 2.1. The microstructural characterisation was carried out by Fourier Transform Infrared (FTIR, from IETcc, Madrid, Spain) spectroscopy using the equipment also mentioned in Section 2.1.

The BSEM/EDX scanning electron microscopy study was performed using an S-4800 scanning electron microscope coupled to a HITACHI energy dispersive X-ray spectrometer at the Eduardo Torroja Institute of Construction Sciences. The samples were embedded in an epoxy resin, cut and polished for observation in BSEM mode.

Thermogravimetric analysis (DTG-TG) of the pastes was carried out using an SDT Q600 (from IETcc, Madrid, Spain). Measurements were carried out from 50 °C to 1000 °C at a rate of 10 °C/min in a nitrogen atmosphere, and platinum crucibles were used for both sample and reference.

Heat flow and total heat were determined by isothermal conduction calorimetry using a TAM AIR thermometric apparatus with 8 dual channels (one for the reference and one for the sample). For this purpose, 20 g of each binder (see Table 3) was mixed with 6 g of distilled water (L/S ratio = 0.3) for 3 min. Then, 5 g of the paste obtained was weighed and placed in the calorimeter, together with a sample of distilled water used as a reference. The tests were carried out at 25 °C ± 1. Due to the curing temperature requirements, 100% alkali cements (AC system) cannot be tested using this technique.

## 3. Results and Discussion

**Figure 4:**
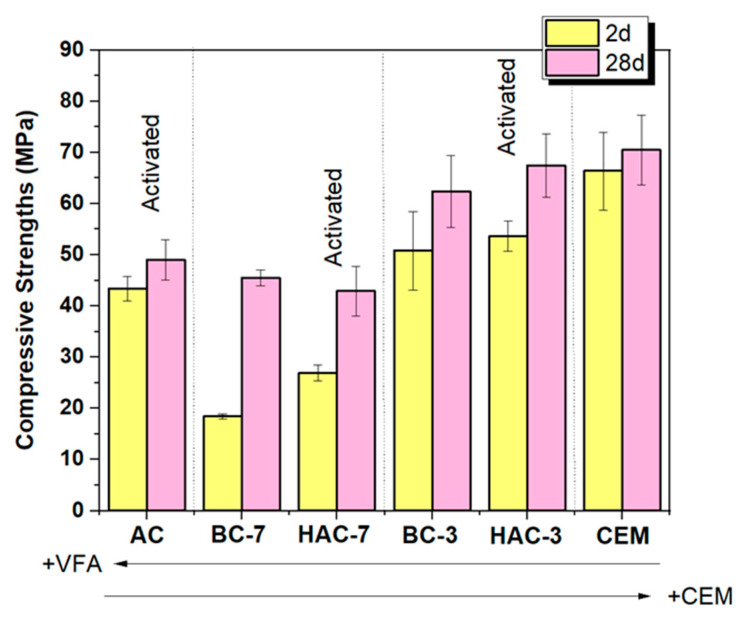
Compressive strengths (MPa) for all pastes: CEM, BC, HAC and AC cements.

Figure 4 depicts the compressive strengths of all the formulated cementitious systems. It can be seen that the CEM paste exceeds 60 MPa at 2 days, with a slight increase in strength at 28 days. Cementitious systems with a high cement content (PC) and about 30% VFA (BC-3 and HAC-3 systems) exhibit strengths of more than 52.5 MPa at 28 days, meeting the resistance values required by the standards for a CEM I. The HAC-3 system, hydrated in the presence of a solid alkali activator, shows slightly higher strengths than BC-3.

On the other hand, the pastes formulated with 70% VFA and 30% PC (BC-7 and HAC-7) show the lowest mechanical strength values. In the case of BC-7, the pozzolanic reaction of VFA is slower than in BC-3 due to the high VFA content and low PC content. In HAC-7, the system with the solid activator, higher strength values are obtained compared to its BC-7 counterpart at early ages, with 2-day strength values of 26.9 ± 1.5 MPa for HAC-7 compared to 18.4 ± 0.4 MPa for BC-7 (without alkali activator). These differences diminish with age, but in both cases, strengths overpass 40 MPa at 28 days, a considerably high value considering the low PC content in these systems.

Finally, the 100% alkali cements (AC) (100% VFA + 8 M NaOH) exhibit compressive strengths above 40 MPa after two days of curing. In this case, the mechanical development progresses slowly over the curing time, reaching 50 MPa at the age of 28 days. At both ages, the developed strengths are significantly lower than those obtained in CEM, BC-3 and HAC-3 but higher than those of BC-7 and HAC-7.

### 3.1. Reaction Products Characterisation

**Figure 5:**
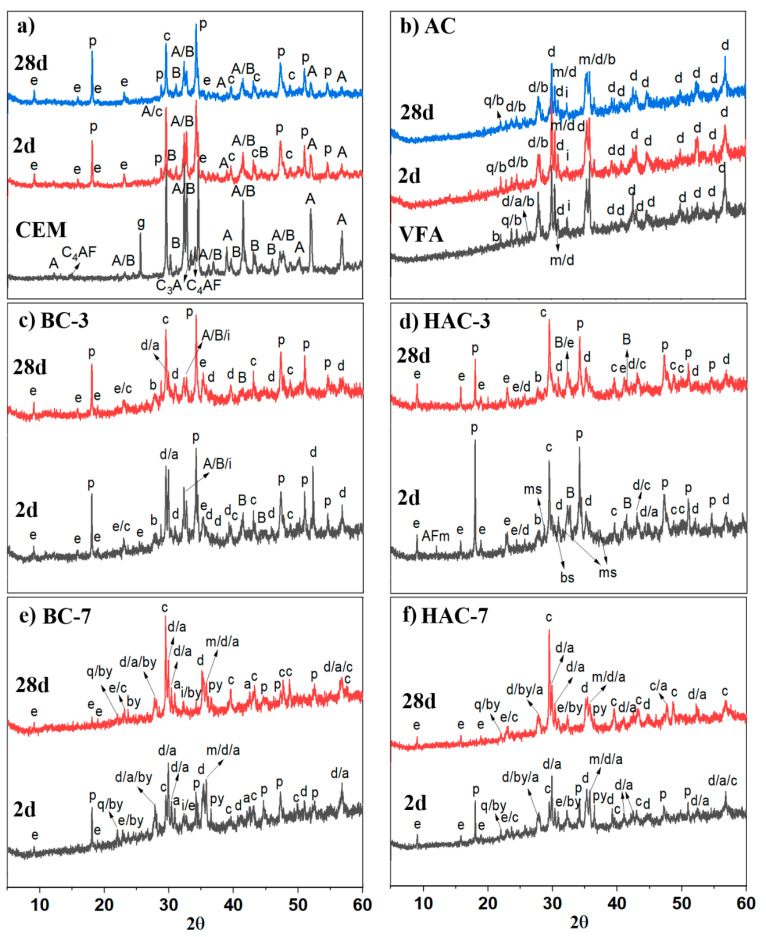
XRD patterns of (**a**) CEM, (**b**) BC-3, (**c**) HAC-3, (**d**) BC-7, (**e**) HAC-7, (**f**) AC after 2 and 28 days of curing. **Legend:** d: diopside (MgCaSi_2_O_6_); a: augite (Ca,Mg,Fe)_2_(Si,Al)_2_O_6_); q: cristobalite (SiO_2_); b: bytownite ((Ca,Na)(Si,Al)_4_O_8_); i: ilmenite (FeTiO_3_); A: alite (3CaO·SiO_2_); B: belite (2CaO·SiO_2_); C_3_A:tricalcium aluminate; e:ettringite (Ca_6_Al_2_(SO_4_)_3_(OH)_12_(H_2_O)_26_) (COD 9015084); p: portlandite (Ca(OH)_2_) (COD 1008780); c: calcite (CaCO_3_); AFm: ((3CaO·Al_2_O_3_·CaSO_4_·12H_2_O) (COD 9013423), bs: basanite (Ca_3_H_3_._6_O_13_._8_S_3_) (COD 9012211), ms: magnesium silicate (MgSiO_3_) (COD 9016052).

Figure 5a shows the diffractograms corresponding to the hydrated cement pastes (CEM) at 2 and 28 days compared to the initial anhydrous PC. CEM shows peaks associated with typical secondary hydration phases, mainly portlandite and ettringite, whose intensity increases with the hydration time. Peaks of calcite, present in the original diffractogram of the anhydrous cement (see Figure 3), are also detected. The intensity corresponding to Alite and Belite peaks decreases with hydration time, and after 2 days, the peaks associated with C_3_A disappear. After 28 days, the peaks corresponding to C_4_AF also disappear.

Figure 5b,c show the diffractograms corresponding to the BC-3 and HAC-3 systems, respectively. In both cements, after 2 days, the crystalline phases diopside and ilmenite, previously identified in the ash, are detected, together with peaks corresponding to anhydrous phases of alite and belite and hydrated phases of cement such as portlandite and ettringite, as well as peaks associated with calcite.

Systems supplemented with an alkaline activator (HAC-3) also show, at 2 days, peaks corresponding to AFm phases and low-intensity peaks associated with bassanite (CaSO_4_-0.5H_2_O), probably residues of added CaSO_4_ as an activator or reaction products obtained from the reaction of portlandite with sulphates added as an activator (see Equation (1)). These peaks disappear at later ages (28 days).

Ca(OH)_2_ + Na_2_SO_4_ → CaSO_4_ + 2NaOH (aq.) (1)

Equation (1). Reaction of solid activator with portlandite.

In addition, in HAC-3, a signal at 2θ of 28.63 is identified, which could correspond to the precipitation of a magnesium silicate not present in the starting materials (PC and VFA) and absent in hydrated CEM.

Figure 5d,e show the diffractograms corresponding to the ash-rich systems BC-7 and HAC-7, respectively. Both systems show very similar diffractograms. After 2 days, the same crystalline phases present in the initial ash (magnetite, cristobalite, diopside, bytownite, augite and pyroxene) are observed due to the high VFA content in these pastes and with greater intensity than in BC-3 and HAC-3. In this case, even after 2 days, there are hardly any peaks corresponding to the anhydrous calcium silicates of the cement (alite and belite). As reaction products of crystalline nature, ettringite and portlandite are detected, although with lower intensity than in BC-3 and HAC-3, due to the low percentage of PC present in these pastes. Peaks associated with calcite are also detected. After 28 days, both systems show slight changes as the signal corresponding to portlandite disappears and those corresponding to calcite increase significantly in intensity. The remaining phases associated with the crystalline components of the ash continue to appear, confirming their low reactivity. In the alkali-activated hybrid systems [30,38,39], the signals associated with ettringite appear slightly higher, which could be explained by the greater number of sulphates available in these systems (in the form of activator).

Finally, in Figure 5f, the diffractograms of volcanic ash pastes activated with 8 M NaOH (AC) are compared with the original ash (VFA). It can be observed that the same crystalline phases identified in the anhydrous volcanic ash are still present: cristobalite, magnetite, diopside, augite, bytownite, ilmenite and pyroxene, indicating is lack of reactivity in an alkaline medium. Due to the amorphous nature of the cementitious gel, the main reaction product of 100% alkali cements based on precursors rich in SiO_2_ and Al_2_O_3_, its identification by XRD is not possible [40]. Crystalline compounds of a zeolitic nature, typically identified in such systems [23,24,25,26], are not detected either.

**Figure 6:**
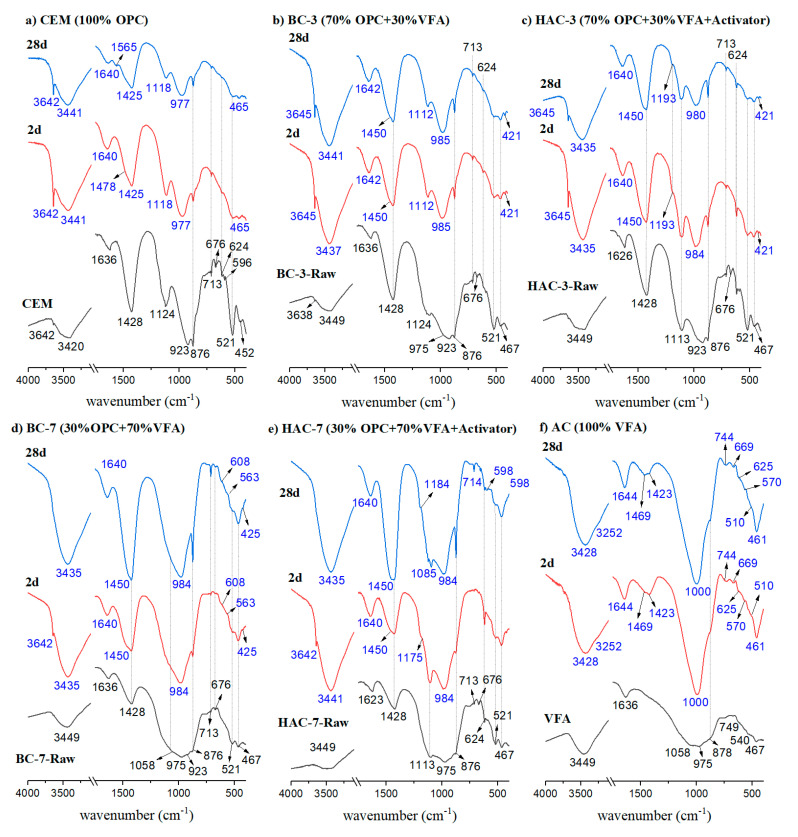
FTIR patterns of CEM, BC-3, HAC-3, BC-7, HAC-7 and AC after 2 and 28 days of curing.

Figure 6 shows the FTIR spectra of different cementitious systems after 2 and 28 days of reaction. In the case of traditional cement pastes (CEM) (Figure 6), clear differences are observed compared to anhydrous cement (PC). Bands associated with clinker phases (calcium silicates at 923, 521 and 452 cm^−1^) disappear, and two bands at 977 cm^−1^ and 465 cm^−1^ appear, attributed to stretching and deformation vibrations of Si-O bonds in the C-S-H gel, respectively [41,42]. The sharp and intense band at 3642 cm^−1^ corresponds to the asymmetric stretching vibrations of O-H bonds in portlandite. The band at 1118 cm^−1^ is assigned to the stretching vibrations of S-O in ettringite [43].

The FTIR spectra at 2 and 28 days for the BC-3 and HAC-3 systems both show differences compared to the FTIR spectra of the original precursors BC3-Raw and HAC3-Raw (70% CEM/30% anhydrous VFA). First, in both pastes, the band at 3640 cm^−1^ is observed, which is assigned to the stretching vibrations of O-H in portlandite. In addition, in both spectra (BC-3 and HAC-3), the main band is located around 985 cm^−1^, a wavenumber characteristic of the asymmetric stretching vibrations of Si-O in C-S-H gel, which, considering the higher proportion of cement compared to VFA in these samples, is probably the dominant cementitious gel in both systems. This band is shifted to higher wavenumber values compared to 100% PC systems (CEM), indicating that C-S-H gels produced in these systems are richer in silicon. Bands of carbonates and remains of crystalline compounds present in the original ash are still identified. Bands of anhydrous calcium silicates from cement disappear. The main difference between both systems (BC-3 and HA-3) is again the intensity of the bands corresponding to sulphates (~1120 cm^−1^), which is more intense in the case of the HAC-3 systems due to the use of Na_2_SO_4_ + 2% CaSO_4_ as solid activators in these pastes.

The spectra of systems with higher ash content (BC-7 and HAC-7) after 2 days show the signal at 3640 cm^−1^ assigned to portlandite, a signal that disappears with hydration/activation time (28 days), in agreement with previous observations made by XRD. The disappearance of this signal could be attributed to various reasons, such as a simple carbonation process (where portlandite reacts with atmospheric CO_2_, precipitating CaCO_3_), as evidenced by the increased intensity of the bands corresponding to the presence of carbonate (1428–1450, 876 and 713 cm^−1^). However, especially in the case of the system supplemented with the alkali activator (HAC-7), it cannot be excluded that portlandite reacts with the solid alkali activator according to Equation (1), reducing its intensity and increasing the intensity of the signal corresponding to sulphates.

In these 70% VFA systems, unlike the previous systems with much higher cement contents, the main band is a broad and asymmetric band located around 1000 cm^−1^. This band would again result from the overlapping of different signals, both from phases corresponding to non-reactive anhydrous ash (crystalline phases and remains of the unreacted vitreous component) and from the reaction products (cementitious gels), which, considering the chemical composition of the starting blends, are probably a mixture of C-A-S-H + (N,C)-A-S-H gels [41,42].

Finally, the FTIR spectrum corresponding to the 100% alkaline AC system (Figure 6) shows significant differences compared to the original VFA anhydrous ash. The main band of VFA with shoulders at 1058 cm^−1^ and 975 cm^−1^ becomes much more intense and sharp, shifting to values around 1000 cm^−1^, a characteristic position for T-O (T: Si or Al) vibrations of N-A-S-H type gel (or more likely (N,C)-A-S-H type gel, considering the CaO content of the starting ash) [41,42]. The band at 460 cm^−1^, corresponding to the δSi-O deformation vibrations of SiO_4_ tetrahedra, also becomes much more intense and sharp. The presence of these bands justifies the formation of a cementitious gel of the N-A-S-H type [25,26,27,28].

**Figure 7:**
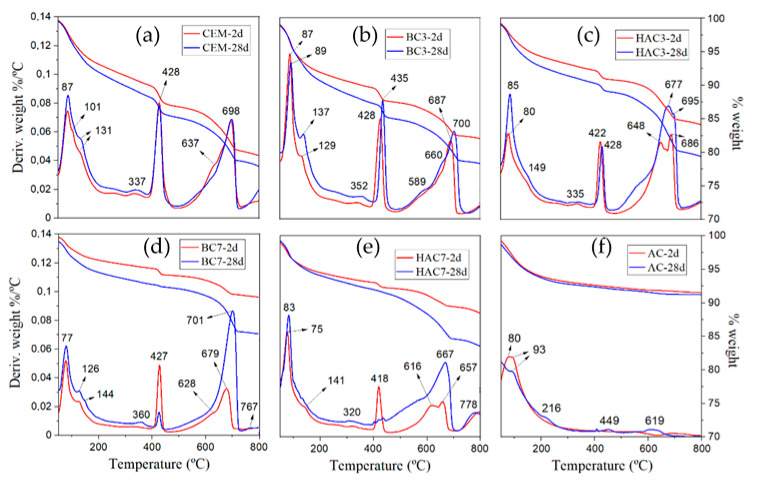
Thermogravimetric analysis of (**a**) CEM, (**b**) BC-3, (**c**) HAC-3, (**d**) BC-7, (**e**) HAC-7 and (**f**) AC after 2 days and 28 days of curing.

Figure 7 shows the TG/DTG curves obtained from thermogravimetric analysis. It is possible to distinguish three temperature ranges associated with mass losses of different reaction products [44,45,46]: (a) Temperatures < 200 °C, corresponding to the loss of free water and the loss of water from hydrates such as C-S-H gel, ettringite (AFt) or (AFm) phases; (b) Between 400 and 450 °C, corresponding to mass losses of water associated with the decomposition of portlandite; (c) Temperature range between 500 and 800 °C, with mass losses of CO_2_ associated with the decomposition of carbonates.

In the 100% cement (CEM) system (Figure 7a), the curves and mass losses between 2 and 28 days are very similar (Table 4). The water loss localised between 60 and 200 °C is associated with the decomposition of C-S-H gels, ettringite and AFm phases. The small signal appearing around 337 °C is associated with the decomposition of small amounts of monosulfoaluminate, while the signal at 428 °C is associated with the dehydroxylation of portlandite. Carbonate decomposition typically occurs between 600 and 800 °C.

The BC-3 system has similar total mass loss values to the reference CEM. In this case, there is a higher mass loss in the 50–200 °C range and a lower mass loss with respect to the portlandite content (see Figure 7, Table 4). However, BC-7 shows significantly lower mass loss in all zones, especially with respect to the portlandite content. In this case, it is evident that the intensity of this peak tends to decrease with hydration time (see Table 4). This phenomenon is due to an overlapping of factors: (i) dilution effect due to less cement (lower initial Ca(OH)_2_ content); (ii) pozzolanic reaction between VFA and portlandite [3,47,48]; and (iii) carbonation of portlandite upon contact with atmospheric CO_2_ [49,50,51], as previously observed in XRD results (Figure 5d,e).

With regard to the HAC systems, it is observed that between 50 and 200 °C, the weight loss increases between 2 and 28 days (see Figure 7), which is associated with the formation of a greater amount of reaction products as the hydration time increases. Another important observation is the lower portlandite content and how it decreases with hydration time, with a more pronounced decrease compared to BC. The reduced presence of portlandite in these systems, compared to their BC counterparts, can be explained by two mechanisms: firstly, part of this portlandite is carbonated (indeed, the carbonate content increases with reaction time), and secondly, part of the portlandite formed during cement hydration reacts with the solid activator (sulphates) (see Equation (1)).

**Figure 8:**
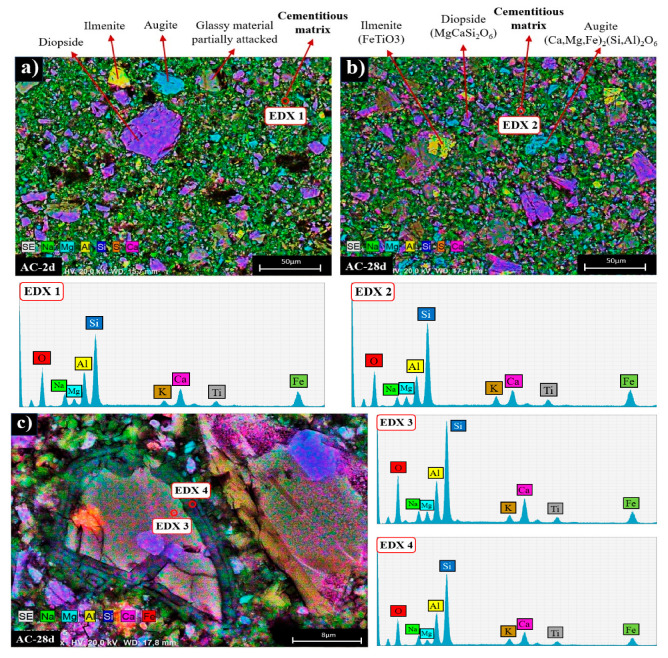
BSEM micrography of (**a**) AC after 2 days of curing (×500), (**b**) AC after 28 days of curing (×500), (**c**) partially attacked VFA particle in AC 28d (×3000).

**Figure 9:**
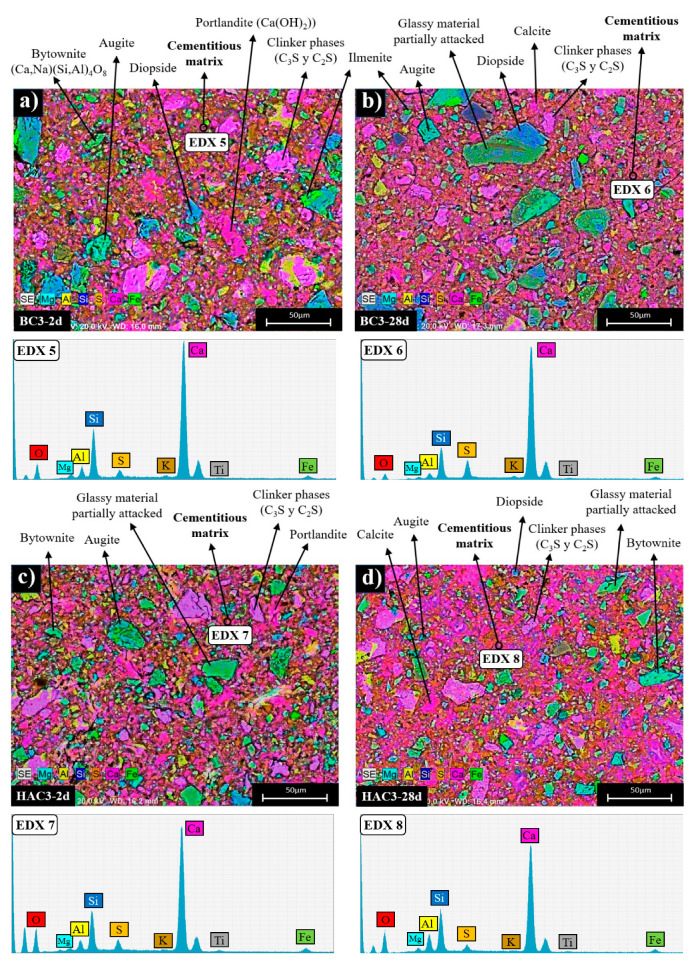
BSEM micrography of (**a**) BC-3 after 2 days of curing (×500), (**b**) BC-3 after 28 days of curing (×500), (**c**) HAC-3 after 2 days of curing, (**d**) HAC-3 after 28 days of curing (×500).

**Figure 10:**
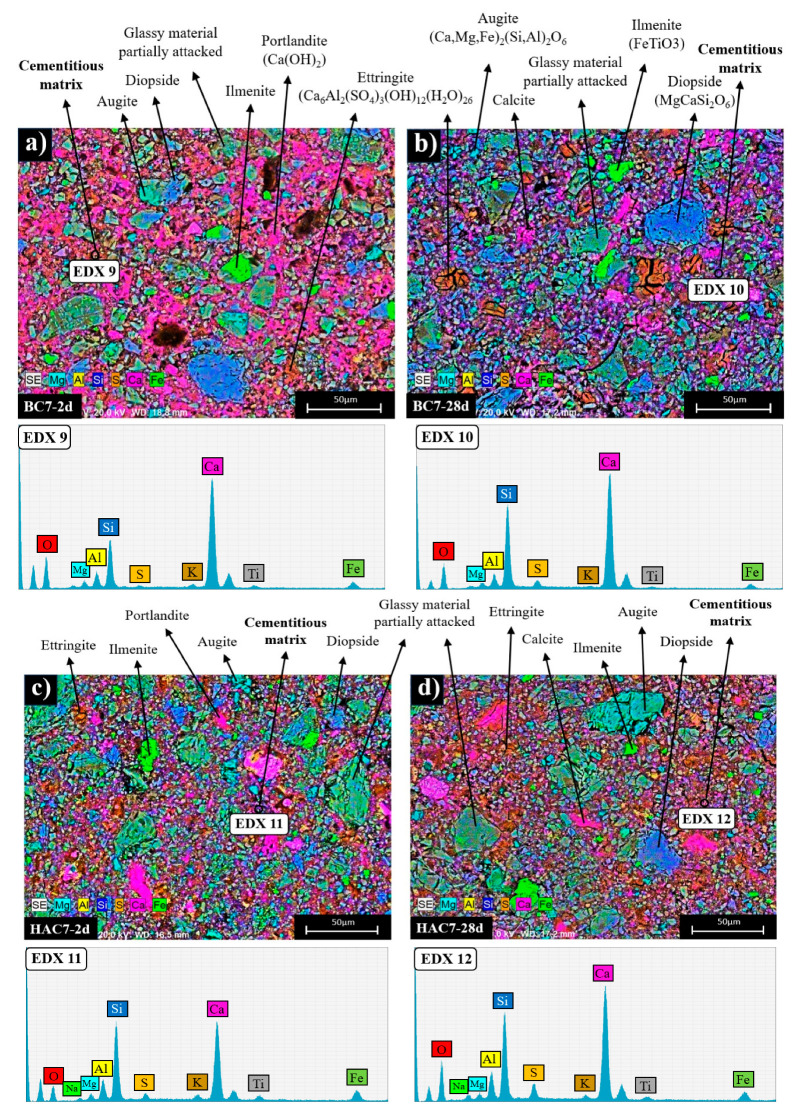
BSEM micrography of (**a**) BC-7 after 2 days of curing (× 500), (**b**) BC-7 after 28 days of curing (× 500), (**c**) HAC-7 after 2 days of curing, (**d**) HAC-7 after 28 days of curing (× 500).

**Figure 11:**
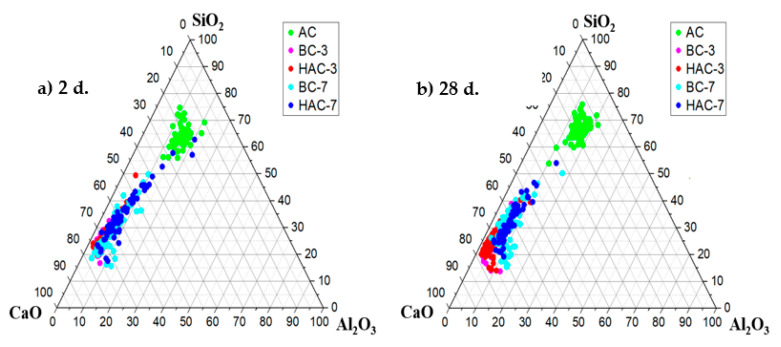
Ternary diagram CaO-SiO_2_-Al_2_O_3_ of elemental EDX analysis of the gel phase.

In order to elucidate the microstructural and compositional changes in the cementitious pastes, the different systems were also analysed by BSEM/EDX (see Figure 8, Figure 9 and Figure 10). Figure 8a,b shows two micrographs corresponding to the mapping of the activated ash at 2 and 28 days (AC system). Both ages show a very similar appearance in which particles associated with the crystalline mineral phases present in the original fly ash (augite, diopside, ilmenite, etc.) can be distinguished, surrounded by a cementitious matrix (EDX analysis 1 and EDX 2). This matrix corresponds to a gel rich in Si and Al [51] and containing Na and Ca in its composition (a (N,C)-A-S-H type gel) (see ternary diagram in Figure 11).

Figure 8c shows a detailed view of a partially attacked ash particle after alkaline activation. There is a compositional difference between the ash particle (EDX analysis 3) and a characteristic halo where the cementitious gel is forming (EDX analysis 4).

Figure 9 shows the maps corresponding to the 30% VFA systems (BC-3 and HAC-3) hydrated at 2 and 28 days. In both systems, the main hydration product identified is a cementitious matrix rich in Ca and Si, with small amounts of Al (EDX analysis 5 and EDX 7), which does not show significant compositional variations at different ages (EDX 6 and EDX 8).

According to the elemental analysis (see ternary diagram in Figure 11), this matrix corresponds to a C-(A)-S-H type gel. In addition, calcite (present in the original cement and/or as carbonation of portlandite upon contact with atmospheric CO_2_) and portlandite are also observed in both systems. Various secondary phases associated with the ash (partially attacked glassy matrix, augite, diopside, ilmenite, etc.) and clinker phases identified by XRD (see Figure 5) are also present. The secondary phases associated with the glassy matrix of the ash appear to be more attacked in the case of the HAC-3 system (Figure 9d) compared to the BC-3 system (Figure 9b). This difference may be attributed to the effect of the alkaline activator in raising the pH of the medium [51].

Figure 10 shows the mapping analyses of systems with approximately 70% VFA, BC-7 and HAC-7. In both systems, similar to the case of cements with 30% VFA (BC-3 and HAC-3), the main hydration product observed is a cementitious matrix rich in Si and Ca, with a lower Al content, corresponding to a C-(A)-S-H type gel (see ternary diagram in Figure 11), together with portlandite and calcite.

At the age of 2 days, the matrix of the system supplemented with the alkaline activator (HAC-7) (Figure 10c) has a higher Si content (EDX 11) than the system without the alkaline activator (BC-7) (Figure 10a), whose matrix is richer in Ca (EDX 9) and has a composition similar to the systems with lower ash content (see ternary diagram Figure 11). This compositional difference in the matrix can be attributed to the effect of the activator on the reactivity of the ashes at early ages, which is more evident in systems with higher ash content [51]. After 28 days, the cementitious matrices in both the activator-supplemented system (Figure 10d) and the non-activator system (Figure 10c) have a similar composition (EDX 10 and EDX 12), enriched in Si and Al compared to systems with lower ash content (see ternary diagram Figure 11).

Another important point to consider is the potential filler effect [52] of unreacted VFA particles (mineral phases). These particles could act as nucleation points favouring the formation of C-S-H or N-A-S-H gels, depending on the type of binder, or even as microfillers improving the mechanical strength of the pastes. This synergistic effect could be responsible for the good compressive strengths obtained in all systems despite the identification of different unreacted VFA particles.

### 3.2. Hydration Kinetics

**Figure 12:**
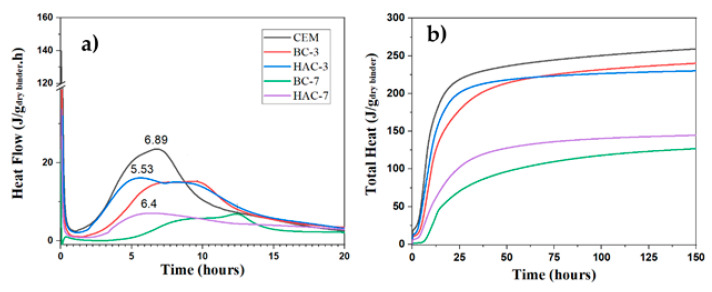
(**a**) Heat Flow (J/g.h) (**b**) Total heat (J/g) for the different cementitious systems (CEM; BC and HAC) (where g represents the grams of binder (VFS+ CEM + Activator).

In order to assess the effect of the activator on the hydration kinetics of the mixtures, an analysis was carried out using isothermal conduction calorimetry. Figure 12a,b show the heat flow curves (J/g.h) and total heat curves (J/g) of the cementitious systems, respectively (excluding the 100% alkaline system, which requires an energy input in the form of heat to facilitate ash dissolution and reaction product precipitation, thereby significantly slowing its reactivity at room temperature).

The heat flow curve of the cement shows the typical stages of the hydration process [53]. After the dissolution peak of the clinker components (not observable due to the initial mixing conditions before the sample was introduced into the calorimeter), there is a short induction period followed by a peak associated with the massive precipitation of hydration products, reaching a maximum at 6.89 h (see Figure 12a). The total heat released by ordinary Portland cement (CEM) reaches 260 J/g (see Figure 12b).

The BC-3 and HAC-3 systems show significant differences compared to the 100% cement system (CEM). In the BC-3 heat flow curve, there is an extension of the induction period, delaying the appearance of the acceleration/deceleration peak (corresponding to the precipitation of reaction products), with the maximum now at 9.60 h (see Figure 12a). The total heat values also decrease slightly compared to the CEM system (see Figure 12b) due to the dilution effect (there is less cement in the system). The presence of the moderately alkaline activator in this system (HAC-3) significantly accelerates the hydration kinetics at early ages, shifting the peak of acceleration/deceleration to shorter times (5.53 h) and introducing a much more pronounced shoulder (around 8.6 h), associated with the precipitation of secondary products such as AFt (it should be noted that this type of cement is supplemented with a mixture of Na_2_SO_4_ + CaSO_4_, providing more available sulphates) [28,51,53,54]. The total heat curve follows a similar trajectory to that of the BC-3 system, although the total heat released by this system is higher up to 50 h, possibly indicating a higher degree of hydration (see Figure 4).

The systems with higher ash content (BC-7 and HAC-7) show slower hydration kinetics due to the higher VFA content and the low PC content (30%). However, it is again shown how the presence of the activator accelerates the hydration kinetics. Both cementitious systems, BC-7 and HAC-7, show lower total heat, which is to be expected considering the high ash content. In particular, the activated system (HAC-7) shows higher total heat than the BC-7 system. This is a clear indication that the activator facilitates both ash dissolution and cement hydration, thereby promoting the precipitation of reaction products. This results in better early strength performance than that observed with BC-7 (see Figure 4).

It is well known that in hybrid cements, the presence of such moderately alkaline activators can react with the portlandite [29,39,55] resulting from cement hydration, producing an in situ alkalinity that would promote an alkaline environment and thereby enhancing the dissolution of the mineral addition, in this case, volcanic fly ash, (see Equation 1). This phenomenon justifies an acceleration of hydration kinetics and improved early strength development.

## 4. Conclusions

The results of this study highlight that volcanic fly ash (VFA) can be used in the production of blended cement (BC), hybrid alkaline cement (HAC) and alkaline cement (AC) at utilisation levels of around 30%, 70% and 100%, respectively. The crystalline phases (diopside, ilmenite, augite, etc.) have a low or negligible degree of reactivity but can be used as fillers. However, the reactive phase associated with the amorphous component of the ash (~50%) reacts in these cements as indicated below:

- In the blended cements (BC-3 and BC-7), volcanic fly ash (VFA) shows pozzolanic behaviour. This effect is more pronounced at higher VFA contents. The incorporation of VFA leads to a reduction in mechanical strength, hydration product formed is a C-(A)-S-H gel, with secondary materials such as AFt and Ca(OH)_2_ detected. The Ca(OH)_2_ content decreases with time due to pozzolanic reaction and carbonation.
- In hybrid alkaline cements (HAC-3 and HAC-7), the use of the alkaline activator accelerates the hydration kinetics and generates a higher degree of heat release, increasing the total heat compared to the same mixtures without the activator (BC-3 and BC-7). This phenomenon can be correlated with a greater precipitation of reaction products. Reaction products include the formation of a C-(A)-S-H gel with higher aluminium contents when higher amounts of VFA are used. The disappearance of Ca(OH)_2_ is related to several factors: (i) less cement leads to less Ca(OH)_2_ formation; (ii) pozzolanic reaction where Ca(OH)_2_ reacts with VFA to form hydration products; (iii) chemical reaction with the activator (see Equation (1)) to produce in situ alkalinity which accelerates the VFA reaction; and eventually (iv) carbonation.
- Alkali-activated cements (100% VFA, AC): The activation of these volcanic fly ashes results in a binder with excellent mechanical properties. The primary reaction product, in this case is a gel of the type (N,C)-A-S-H, similar to that obtained when other types of fly ash are activated.

## Figures and Tables

**Table 1:** Chemical composition (% wt.) of VFA and PC.

	SiO_2_	Al_2_O_3_	Fe_2_O_3_	CaO	MgO	TiO_2_	Na_2_O	K_2_O	P_2_O_5_	SO_3_	Others	LoI *
VFA	41.56	14.49	14.73	11.92	5.57	4.03	4.21	1.75	0.81	0.19	0.74	-
PC	18.13	4.29	3.00	61.47	3.33	0.26	0.50	0.56	0.10	3.00	5.36	4.36

* Loss on ignition 1000 °C.

**Table 2:** CaO saturated solution test and HF attack analysis results of the VFA.

**CaO Saturated Solution Test Results**					
**Age**		**pH**		**% CaO Fixed**	
2		12.51 ± 0.01		13.46 ± 0.05	
28		12.32 ± 0.01		55.48 ± 3.16	
**HF Attack Analysis Results**					
**%SiO_2_**		**%Al_2_O_3_**		**SiO_2_ + Al_2_O_3_** **%Reactive**	**Ratio** **SiO_2_/Al_2_O_3_**
**Initial**	**Reactive**	**Initial**	**Reactive**		
41.56	35.73	14.49	10.70	46.43	3.34

**Table 3:** Dosification of pastes (% wt.).

Name	BINDERS (B)		Activator	Liquid Hydration (L)	L/B	Curing Conditions
	VFA	PC				
CEM	--	100	--	Water	0.3	20 h. 25 °C
BC-3	30	70	--	Water	0.3	20 h. 25 °C
HAC-3	30	70	SAc *	Water	0.3	20 h. 25 °C
BC-7	70	30	--	Water	0.3	20 h. 25 °C
HAC-7	70	30	SAc *	Water	0.3	20 h. 25 °C
AC	100	--	LAc *	NaOH 8 M	0.4	20 h. 85 °C

* SAc: Solid Activador 3% Na_2_SO_4_ + 2% CaSO_4_ (%wt.) relative to binder; * LAc: Liquid Activador, NaOH 8 M solution.

**Table 4:** Portlandite and carbonates % by weight calculated from TG/DTG curves.

Sample	% Portlandite		% Carbonates	
	2 d	28 d	2 d	28 d
**CEM**	10.27	9.86	16.36	16.36
**BC-3**	7.81	8.22	12.04	14.77
**HAC-3**	5.34	4.93	15.00	21.36
**BC-7**	4.11	1.64	7.27	15.22
**HAC-7**	3.28	2.46	8.18	16.36

## Data Availability

Data are contained within the article.
